# Optimizing the Mechanical Properties of Ultra-High-Performance Fibre-Reinforced Concrete to Increase Its Resistance to Projectile Impact

**DOI:** 10.3390/ma14175098

**Published:** 2021-09-06

**Authors:** Anna L. Mina, Konstantinos G. Trezos, Michael F. Petrou

**Affiliations:** 1 Laboratory of Reinforced Concrete, School of Civil Engineering, National Technical University of Athens, 5 Iroon Polytechniou Street, 15773 Zografou, Greece; ctrezos@central.ntua.gr; 2 Department of Civil and Environmental Engineering, University of Cyprus, 75 Kallipoleos Avenue, P.O. Box 20537, Nicosia 1678, Cyprus; petrou@ucy.ac.cy

**Keywords:** ultra-high-performance concrete, ultra-high-performance fibre-reinforced concrete, stress–strain curves, compressive strength, direct tensile strength, indirect tensile strength, modulus of elasticity, Poisson’s ratio, finite elements

## Abstract

This study describes an extensive experimental investigation of various mechanical properties of Ultra-High-Performance Fibre-Reinforced Concrete (UHPFRC). The scope is to achieve high strength and ductile behaviour, hence providing optimal resistance to projectile impact. Eight different mixtures were produced and tested, three mixtures of Ultra-High-Performance Concrete (UHPC) and five mixtures of UHPFRC, by changing the amount and length of the steel fibres, the quantity of the superplasticizer, and the water to binder (*w*/*b*) ratio. Full stress–strain curves from compression, direct tension, and flexural tests were obtained from one batch of each mixture to examine the influence of the above parameters on the mechanical properties. The Poisson’s ratio and modulus of elasticity in compression and direct tension were measured. Additionally, a factor was determined to convert the cubic strength to cylindrical. Based on the test results, the mixture with high volume (6%) and a combination of two lengths of steel fibres (3% each), water to binder ratio of 0.16% and 6.1% of superplasticizer to binder ratio exhibited the highest strength and presented great deformability in the plastic region. A numerical simulation developed using ABAQUS was capable of capturing very well the experimental three-point bending response of the UHPFRC best-performed mixture.

## 1. Introduction

Ultra-High-Performance Concrete (UHPC) is a new type of concrete that demonstrates high compressive strength and durability compared to conventional concrete. The high strength is achieved due to the use of a superplasticizer, increased percentage of microsilica, low water to binder, where the binder is cement plus microsilica, (*w*/*b*) ratio, and lack of coarse aggregate [1,2,3,4]. The major disadvantage of high strength concrete is the lack of ductility. Steel fibres with high tensile strength are added to overcome this disadvantage. The addition of fibres also provides a strength increase and cracking control. When steel fibres are included in the matrix, Ultra-High-Performance Fibre-Reinforced Concrete (UHPFRC) is formed. At first, it was called Reactive Power Concrete and developed in France [5].

UHPFRC has many applications in the construction industry [6,7,8], being used in bridges and high-rise buildings. A significant amount of research has been conducted on UHPFRC composition in order to improve its mechanical properties. Several research teams have experimented with altering the quantity of surfactants, type of cement, curing methods, quantity of water, fibre content, and type of fibres, investigating their effects on durability, workability, shrinkage, and mechanical properties [9,10,11,12,13,14,15,16]. The compressive strength of cubes and flexural tensile strength are the mechanical properties most often measured [10,11,12,13]. Kazemi and Lubell [11] determined the stress–strain response under compressive and direct shear loading, for different fibre contents and specimen sizes. Moreover, the interaction between steel fibres and the matrix in UHPFRC attracted the attention of many researchers who studied the direct tensile behaviour of UHPFRC [16,17,18,19,20,21]. Graybeal and Baby [18] presented a new test method to capture the full stress–strain curve in direct tension. Wille et al. [19] presented an extensive review of tensile test setups used by other researchers and proposed a modified testing setup that was adopted by Pyo et al. [20]. Yoo et al. [21] studied the effect of the steel fibre type on the tensile behaviour of UHPFRC. Only a few studies captured the full stress–strain curves from both uniaxial compression and direct tension due to the experimental difficulties of recording uniaxial compressive and direct tensile strains [22]. In the present study, the direct tension test was performed in order to capture the full stress-strain curve including the plastic zone. The splitting tensile test provides only tensile ultimate strength and also the cylinder specimen is exposed to a complex combination of tension, shear, and compression, along with a considerable stress gradient. This means that the resulting tensile strength could be inaccurate [22]. Full stress–strain curves are necessary to appreciate the performance of an UHPFRC, due to its superior postcracking behaviour.

Current structures are not designed to resist terrorist attacks, which is crucial due to the recent increase in such attacks. The present research aims to optimize the design of UHPFRC to provide the mixture with the highest resistance to bullet impact loading. A similar mixture was developed in a previous study [13] and tested under blast and impact loads [23], but further investigation is needed. Researchers noted that, generally, concrete members with higher compressive strength provide improved resistance to penetration [24,25]. The penetration process begins when the kinetic energy of the bullet is transferred to the target after the hit. The target is exposed, at the same time, to compression on the front face and tension on the rear face. According to O’Neil et al. [26], resistance to the projectile penetrating through a material is a measure of toughness—the energy needed to crack the matrix in tension. Zhang et al. [25], in an experimental study, showed that a low penetrating depth can be achieved with a compressive strength over 150 MPa and a decrease in crater diameter and spalling can be obtained with the addition of steel fibres. With the addition of steel fibres, the crater region is confined to a localized area [25]. Sovjak et al. [27,28] proved through extensive research that the fracture energy increases when the aspect ratio and volume fraction of fibre increase. Higher fracture energy results in higher energy absorption and better resistance to projectile impact. The influence of steel fibre volumes of 1%, 2%, and 3% [29] and 0.125%, 0.25%, 0.5%, 1%, and 2% [30] on the mechanical properties, penetration depth, and crater diameter was studied, but only with one type of steel fibre, 13 mm long. A combination of two types of fibres, specifically 1.5% per volume long length and 0.5% per volume short length achieved the highest compressive and flexural strength [12]. Based on the literature review, UHPFRC designed for projectile impact loading must have high tensile and compressive strength and exhibit ductile behaviour with a plastic region.

The scope of this paper is to present the results of an experimental investigation to optimize UHPFRC in order to achieve high strength and ductility, which are essential for resisting projectile impact. Eight mixtures are tested in this study to investigate the influence of the quantity of steel fibres, combination of different lengths of steel fibres, the water to binder ratio, and the superplasticizer to binder ratio on the mechanical properties of UHPFRC. The compressive strength, direct tensile strength, flexural tensile strength, modulus of elasticity, and Poisson’s ratio from compression and direct tension are measured. Moreover, for all mixtures, complete stress–strain curves in compression, direct tension, and flexural are determined and presented in this paper. It is important to mention that it is difficult to find full stress–strain curves from all three tested UHPFRC mixtures in the literature. Additionally, we present a finite element model developed in ABAQUS using the material properties obtained in this research. The concrete damage plasticity model in ABAQUS is used for the numerical simulation of a three-point flexure test and the predictions are compared to the experimental results.

## 2. Experimental Program

### 2.1. Mix Design

The mix design that was used in this study is based on a previous study by Benson and Karihaloo [31], which was modified by Nicolaides et al. [13] in order to use materials from the local market. This mix design was the first UHPFRC mixture in Cyprus and was developed during an extensive experimental investigation aiming to select the best constituent materials and curing procedure to improve workability and mechanical properties, measured using cube compressive strength and indirect tension (three-point bending) tests.

For this study, eight mixtures were developed and tested: Three UHPCs without steel fibres and five UHPFRCs with steel fibres. The objective was to further improve the previously selected design in order to achieve the best performance under impact loading conditions. Two types of steel fibres were used, 6 mm long with 0.16 mm diameter and 13 mm long with the same diameter. Two of the five mixtures included only one type of steel fibres (13 mm long) and the other three included a combination of two lengths of steel fibres. The mixture proportions were the same with changes in the quantity of superplasticizer, the percentage by volume and length of the steel fibres, and the water/binder ratio. The superplasticizer to binder ratio varied from 0.045 to 0.061, water to binder ratio decreased from 0.20 to 0.16, and the percentage of steel fibres increased from 0% to 2% and 6%. The mixtures with 2% by volume steel fibres contained only the 13-mm-long fibres. The mixtures with 6% by volume fibres had both lengths of steel fibres, with 5-1 and 3-3 percentage of short (6 mm) to long (13 mm). The names of the different mixture designs are represented as follows: a(a1,a2)/b/c where a, a1, a2, b, and c are the percentage (%) of the fibres by volume, percentage (%) by volume of the 6-mm-long fibres, percentage (%) by volume of the 13-mm-long fibres, percentage (%) of the water to binder ratio, and the percentage (%) of the superplasticizer to binder ratio, respectively. Table 1 and Table 2 feature the details of the mixture proportions.

The materials used to produce the different mixtures are presented below:

- Portland Cement CEM I strength with a Clinker content of 95% and strength category 52.5R;
- Microsilica with a silica content (SiO_2_) 97%;
- Polycarboxylate polymer-based superplasticizer;
- Calcareous sand from a local quarry, grading 125–250 μm and 250–500 μm;
- Steel fibres with 0.16 mm diameter and 6 mm or/ and 13 mm length. Density of 7850 kg/m^3^ and tensile strength of 3000 MPa.

The following mixing procedure was implemented based on the studies by Benson and Karihaloo [31]. Sand, silica fume, and Portland cement were mixed first. Steel fibres were added in three different dosages with 2 min of mixing time for each dosage after they passed from a vibrating sieve. Two-thirds of the superplasticizer was added to the water. The created liquid was divided into three parts: One-half, one-quarter, and one-quarter. These parts were added continuously in the dry mixture with a 2-min stirring time for each part. Finally, the remaining one-third of the superplasticizer was added directly to the mixture. All of the mixtures were made using the same equipment in order to eliminate any variations in the results.

### 2.2. Specimens and Curing

All the specimens for each mixture were produced from the same batch and based on the CYS EN 12390–1:2009 [32] standard. They were cast into moulds while they were compacted on a vibrating table. Table 3 presents the number and dimensions of each type of specimens.

The curing of the specimens was conducted based on a study by Nicolaides et al. [13]. Twenty-four hours after casting, specimens were demoulded and placed in a water tank where the temperature of the water was gradually raised from 20 to 90 °C (3rd day) and held at 90 °C for 11 days. On the 13th day, the temperature began to decrease back to 20 °C (14th day), while the specimens remained in water at a temperature of 20 °C until testing.

### 2.3. Experimental Tests

The compressive and flexural tensile tests (three-point bending test) were executed using a hydraulic testing machine, which included a 5000 kN compression frame and a 250 kN flexural frame. Direct tensile tests were performed using a 300 kN materials testing machine. Table 4 shows the test that was conducted for each type of specimen.

#### 2.3.1. Uniaxial Compression Test

**Figure 1:**
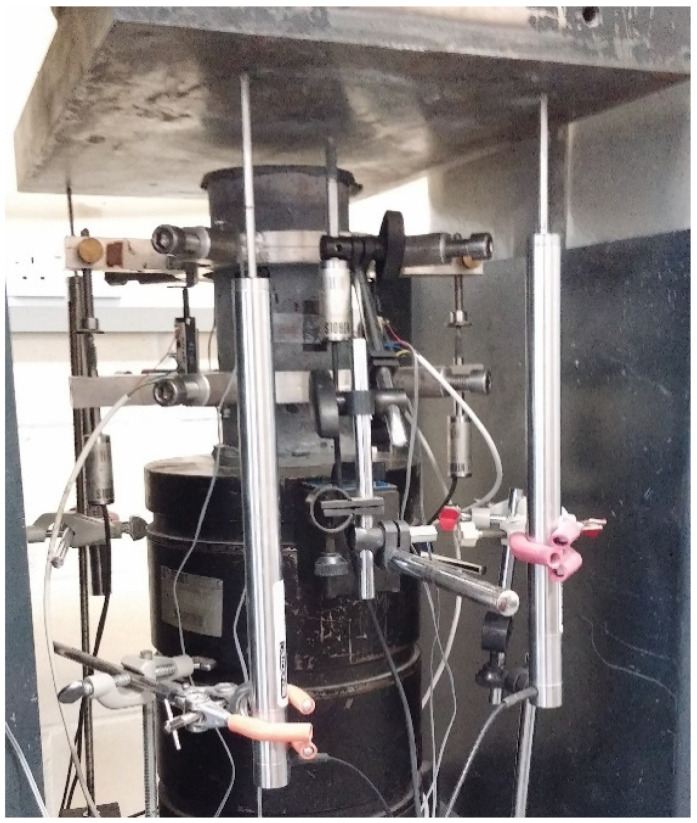
Set-up of uniaxial compression test.

**Figure 2:**
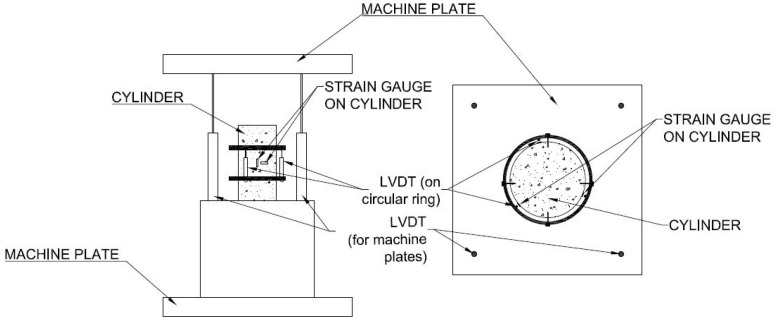
Uniaxial compression test-sketch of the instrumentation set-up.

Cubes with dimensions (100 mm × 100 mm × 100 mm) were tested under compression according to CYS EN 12390—3:2009 [33], at a rate of 0.5 MPa/s. In the uniaxial compression testing of cylindrical specimens (d = 100 mm, h = 200 mm), two circular rings were mounted around the specimen and fixed with four screws. The rings were horizontal with approximately 70 cm between them and symmetrical to the specimen’s mid-height. On the rings, three LVDTs were attached at a 120° angle between them to be able to measure the occurring uniaxial displacements and capture the full stress–strain curve. Additionally, at the surface of the specimen at the mid-height, three longitudinal with 30 mm and 60 mm length strain gauges were attached on the specimen again at a 120° angle and one transverse with 10 mm length strain gauge (Figure 1 and Figure 2), in order to determine the modulus of elasticity and Poisson’s ratio. The strain gauges measurements were verified by LVDT measurements. Cylinders were capped according to the ASTM C 617-98 (2003) [34] standard using a sulphur mortar. Both surfaces, top and bottom, of the cylindrical specimens were capped to ensure similar conditions during the test. In order to ensure that the machine plates moved in parallel to each other, four LVDTs were placed on the machine loading plate. Compression tests were performed using the displacement control with a displacement rate of 0.015 mm/min.

#### 2.3.2. Direct and Indirect Tension Test

**Figure 3:**
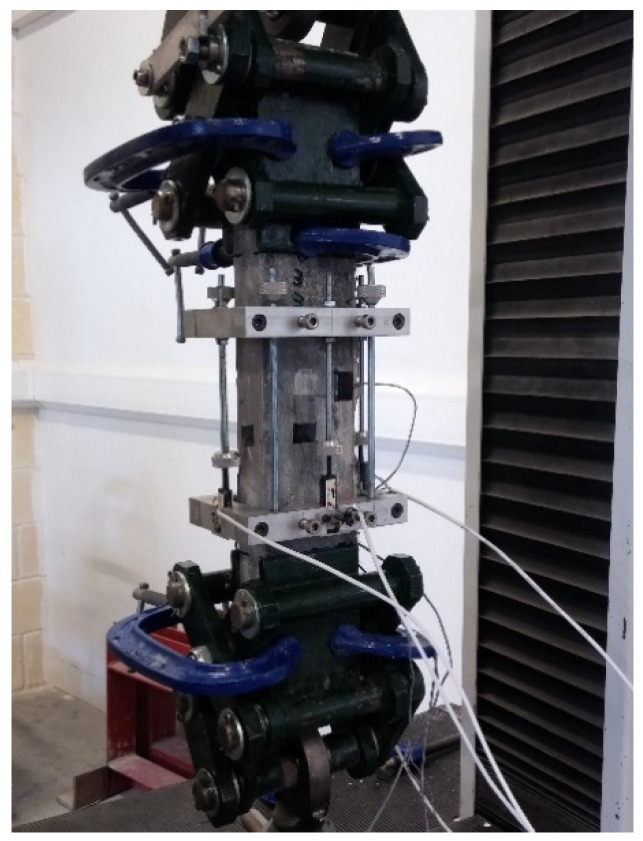
Set-up of direct tension test.

**Figure 4:**
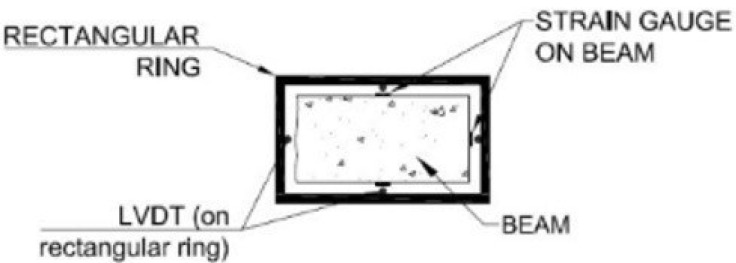
Direct tension test-sketch of instrumentation of set-up.

The aim was to use a setup for direct tension without the complexity of constructing dogbone specimens. The technique was adopted by Kolias and Williams [35]. The specimens were rectangular cross-section beams. A system of special stainless-steel grippers with a hinged head was prepared. On the surface of the specimens, three longitudinal with 30 mm and 60 mm length and one transverse with 10 mm length strain gauges were attached. Two rectangular rings were fixed to the specimen with four screws. The rings supported four LVTDs, one at each side of the specimen (Figure 3 and Figure 4). The strain gauges measurements were verified by LVDT measurements. The displacement rate for the direct tension tests was 0.04 mm/min. The fibre distribution depends on the shape and size of the specimens and, according to Soroushian and Lee [36], “the fibre orientation seems to approach 3-D condition gradually as the cross-section dimensions exceed two times the fibre length.” Based on the above knowledge, the minimum dimension of the beams was selected to be more than two times the length of the longer fibres (h = 100 mm and b = 50 mm). For mixture 6/16/6.1, dogbone-shaped specimens with smaller thickness were also cast for verifying the effects. The middle section of the dogbones was h = 40 mm and b = 55 mm.

**Figure 5:**
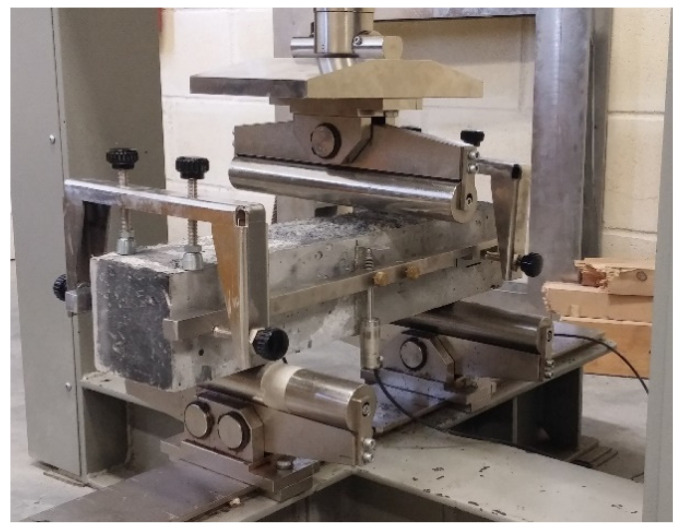
Set-up of flexural tension test.

Six of the mixtures were also tested in indirect tension (three-point bending) tests using prismatic beams according to EN 12390—5:2009 [37] (Figure 5).

## 3. Results and Discussion

### 3.1. Mechanical Properties

Table 5 presents the results (average values from each mixture) for the compressive strength of cubes and cylinders, direct and indirect tensile strength, modulus of elasticity, and Poisson’s ratio. 

The ultimate compressive strength values of cubes:

- Increased by 25.6% when long fibres 2% (2(0-2)/16/6.1) by volume were included in mixture 0/16/6.1.
- Decreased, when the *w*/*b* ratio increased from 0.16 to 0.20, by 15.8% in mixtures without fibres (0/16/6.1 vs. 0/20/6.1) and by 18.0% in mixtures with 2% by volume of fibres (2(0-2)/16/6.1 vs. 2(2-0)/20/6.1).
- Increased by 13.4% when fibres increased from 2% (2(0-2)/16/6.1) to 6% (5-1) ratio 5:1 of 6 mm short fibres to 13 mm long (6(5-1)/16/6.1).
- Increased by 6.2% when in mixture 6(5-1)/16/6.1, with 6% (5-1) by volume of fibres equal amount of short and long fibres (3% by volume, respectively) was placed (6(3-3)/16/6.1).
- Increased by 7.8% when the superplasticizer per binder increased from 0.045 to 0.061 in mixtures with 6% (5-1) by volume of fibres (6(5-1)/16/4.5 vs. 6(5-1)/16/6.1).

The ultimate tensile strength from direct tensile tests:

- Increased by 109.0% when long fibres 2% (2(0-2)/16/6.1) by volume included in mixture 0/16/6.1 and 70.3% when long fibres 2% (2(0-2)/20/6.1) by volume were included in mixture 0/20/6.1.
- Decreased when *w*/*b* increased from 0.16 to 0.20 by 15.9% for mixtures without fibres (0/16/6.1) vs. 0/20/6.1) and 31.5% at mixtures with 2% by volume (2(0-2)/16/6.1 vs. 2(0-2)/20/6.1).
- Exhibited a slight increase of 3.6% when fibres increased in mixture 2(0-2)/16/6.1 from 2% (0-2) to 6% (5-1) fibres by volume (6(5-1)/16/6.1).
- Decreased by 31.6% in beam specimens, when in mixture 6(3-3)/16/6.1 with 6% by volume of fibres equal amount of short and long fibres (3% by volume, respectively) rather than mixture 6(5-1)/16/6.1 with 6% and 5% short fibres and 1% long fibres. Increased by 11.6% when bone specimens were used for mixture 6(3-3)/16/6.1 compared to mixture 6(5-1)/16/6.1.

The ultimate tensile strength from flexural tensile tests:

- Showed a significant increase of 100% when 2% (2(0-2)/16/6.1) by volume fibres included in mixture 0/16/6.1 and 280% when fibres 2% (2(0-2)/20/6.1) by volume were included in mixture 0/20/6.1.
- Increased by 73.7% in mixtures without fibres (0/16/6.1 vs. 0/20/6.1) and minor decreased from 29.0 to 26.6 MPa (8.3%) in mixtures with 2% by volume of fibres (2(0-2)/16/6.1 vs. 2(0-2)/20/6.1), when the *w*/*b* ratio decreased from 0.20 to 0.16.
- Increased by 19.2% when fibres increased in mixture 2(0–2)/16/6.1 from 2% (0–2) to 6% (5-1) (6(5-1)/16/6.1). When equal numbers of short and long fibres (3% by volume) were placed in mixture with 6% by volume of fibres (6(3-3)/16/6.1), it remained almost the same (slight improvement of 5%) as 6(5-1)/16/6.1.

**Figure 6:**
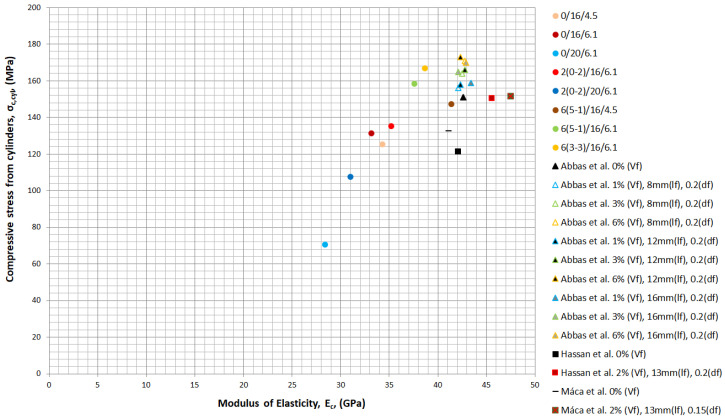
Comparison with literature of compressive stress from cylinders (σ_c,cyl_) and modulus of elasticity (E_c_).

The modulus of elasticity was calculated from the plot of the stress-strain curve. It was calculated from the region between 25% to 65% of the ultimate load in order to exclude the low values near zero and the high values where the plastic zone begins. This ensures that we are in the elastic zone. The modulus of elasticity in compression was lower than in direct tension (Table 5). In Figure 6, a comparison is presented between the experimental results from the literature and the experimental results from this research. Average values from each mixture are presented. All modulus of elasticity values from experimental results were lower than the values obtained from the literature. Abbas et al. [38], with 1%, 3%, and 6% per volume steel fibres, measured a modulus of elasticity around 43 GPa; Hassan et al. [22] and Maca et al. [17], using mixtures with 2%, had values that varied from 46 to 48 GPa. In contrast to the aforementioned literature results, the modulus of elasticity measured in this study for 2% per volume steel fibres varied from 31 to 35 Gpa and for 6% by volume steel fibres was around 40 GPa. Local material properties could be responsible for the low values [13]. This interpretation, however, needs further investigation. The Poisson’s ratio was calculated from the plot of the longitudinal strain vs. transverse strain curve. It was calculated from the region between 25% to 65% of the ultimate load in order to exclude the low values near zero and the high values where the plastic zone begins. This ensures that we are in the elastic zone. UHPC and UHPFRC mixtures had higher values of Poisson’s ratio, which ranged from 0.24 to 0.27 with an average of 0.25 compared to normal concrete expected values between 0.15 and 0.20. 

**Figure 7:**
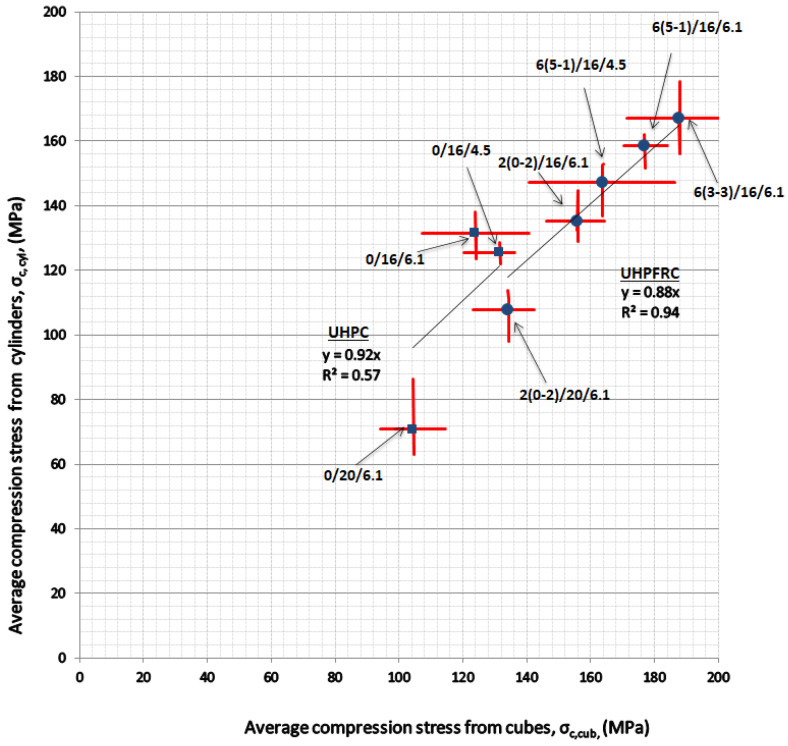
Correlation of compressive strength from cubes and cylinders.

Figure 7 presents the ultimate compressive strength for the two types of specimens from each mixture. The red lines represent the range of the values and the dots the average from all specimens (see Table 3). For normal concrete, the cylindrical compressive strength is lower than the cubic strength. A factor of approximately 0.85 is usually used to convert cubic strengths to cylindrical strengths, for cubes with specimen side 150 mm and cylinders with diameter 150 mm and height 300 mm. The factor increases when the strength increases. The results presented here for UHPFRC with 2% and 6% of steel fibres give a factor of 0.88 with *R^2^* = 0.94 (the R-squared value of the correlation). A factor equal to 0.96 for UHPFRC was reported in the literature [39], but with a smaller specimen size (the cube’s height was 70.7 mm and the cylinder’s diameter was 76 mm). For UHPC, the reduction factor was 0.92, but the *R^2^* was low at 0.57.

### 3.2. Full Stress–Strain Curves from Uniaxial Compression Tests and Parametric Investigation

**Figure 8:**
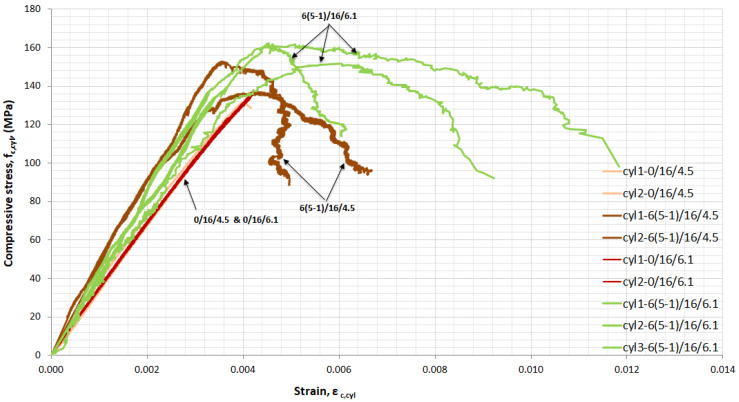
Stress–strain curves in compression for UHPC and UHPFRC 5(5-1)-influence of superplasticizer.

**Figure 9:**
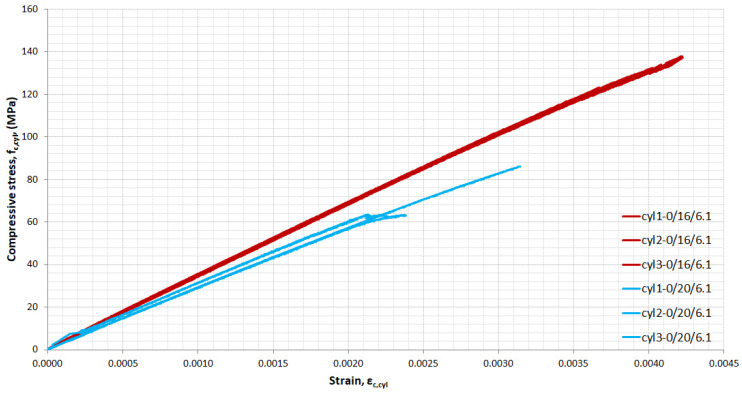
Stress–strain curves in compression for UHPC: The influence of *w*/*b*.

**Figure 10:**
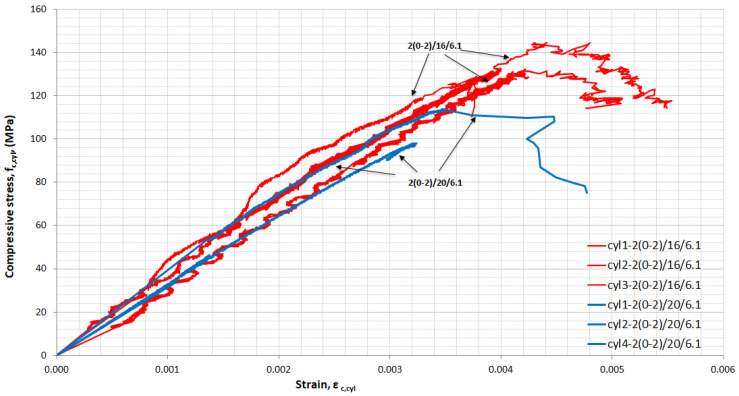
Stress–strain curves in compression for UHPFRC 2(0-2): The influence of *w*/*b*.

**Figure 11:**
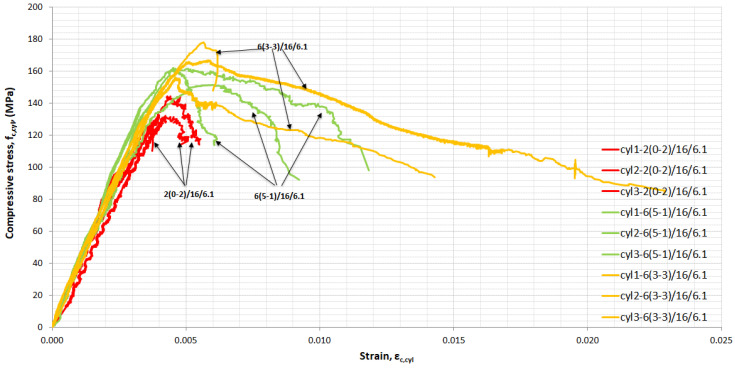
Stress–strain in compression for UHPFRC-influence of fibre content and length.

For each mixture, cylinders were tested in uniaxial compression and full stress–strain curves were obtained. Figure 8, Figure 9, Figure 10 and Figure 11 show the influence of superplasticizer, *w*/*b*, quantity, and length of fibres on the elastic and plastic phase of the curve. All of the cylinders from each mixture are included in the figures. 

Two percentages of superplasticizer to binder were used, 4.5% and 6.1%. In Figure 8, the influence of the quantity of superplasticizer for UHPC mixtures without fibres and UHPFRC with 6% (5-1) fibres by volume is presented. The increase in the quantity of superplasticizer for mixtures with 6% (5-1) fibres by volume offered better workability during the cast. For UHPFRC 6% (5-1), the modulus of elasticity decreased from 41.4 to 34.2 GPa and the ultimate compressive strength increased by 7.5% when more quantity of superplasticizer was added. 

Figure 8 shows that UHPFRC reached a higher range of strain when the amount of superplasticizer increased. It seems that the addition of superplasticizer helped achieve a uniform distribution of fibres and the homogenization of the mixture in the specimen and consequently helped reduce the length/width of the cracks and develop multicracking with stress maintained and high strain taking place. 

For UHPC (Figure 8), the superplasticizer had a limited effect on the elastic phase and the ultimate compressive strength increased by 4.8%, the modulus of elasticity decreased by 3.2%, and, as the first crack appeared, the specimen exhibited its ultimate tensile strength and failed in a sudden, explosive manner.

Figure 9 and Figure 10 demonstrate the effect of the increase of *w*/*b* ratio from 0.16 to 0.20 on the compression behaviour of UHPC and UHPFRC 2(0-2). As expected, when the amount of water increased in UHPC, the compressive strength and modulus of elasticity decreased (Figure 9). The corresponding strain for ultimate compressive stress decreased by 40.5% when *w*/*b* increased from 0.16 to 0.20. In contrast, when the amount of superplasticizer decreased, the decrease in corresponding strain was only 2.8%. Sudden failure occurred for all the specimens produced without fibres. In the UHPFRC 2(0-2)/20/6.1 mixture, one of the three cylinders exhibited nonlinear behaviour (Figure 10) while in mixture 2(0-2)/16/6.1, two of the three cylinders achieved nonlinear region. Increasing the amount of water from *w*/*b* = 0.16 to 0.20 in the mixtures of UHPFRC with 2% (2-0) by volume fibres significantly reduced the compressive strength of the cylinders by 20.5% and the corresponding strain at maximum stress from 4.3 × 10^−3^ (on average) to 3.4 × 10^−3^ (on average), which corresponds to a 21% reduction.

In Figure 11, the influence of the quantity and length of fibres is presented. The mixture with 2% by volume 13-mm long fibres reached 135.3 MPa cylinder strength on average and a corresponding strain equal to 4.3 × 10^−3^. When the percentage of fibres was increased to 6% by volume, of which 5% were short 6-mm fibres and 1% were long 13-mm fibres, the cylindrical strength increased by 17.1%, with an ultimate strength of 158.4 MPa. The strain at ultimate strength was 5.6 × 10^−^^3^. Replacing 2% of 6-mm-long fibres with 13-mm-long fibres in mixtures with 6% by volume, 6% (3-3) contributed significantly to the development of larger deformations in the plastic region (Figure 11). The replacement of 2% 6-mm-long fibres’ contribution to compressive strength is low and equal to 5.5% for cylindrical specimens.

**Figure 12:**
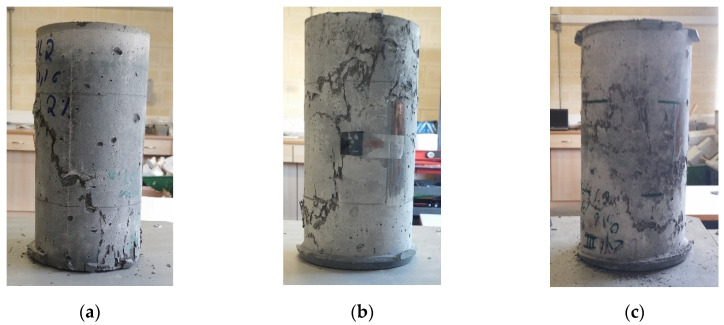
Failure of UHPFRC mixtures (**a**) 2(0-2)/16/6.1, (**b**) 6(5-1)/16/6.1, and (**c**) 6(3-3)/16/6.1.

Figure 12 shows the UHPFRC specimen’s failure condition. Failure of mixture 2(0-2)/16/6.1 (a) shows wider cracks, in contrast to mixtures with 6% by volume steel fibres (b and c). Similar failure types are reported in the literature [22].

### 3.3. Full Stress–Strain Curves from Direct Tension Tests and Parametric Investigation

**Figure 13:**
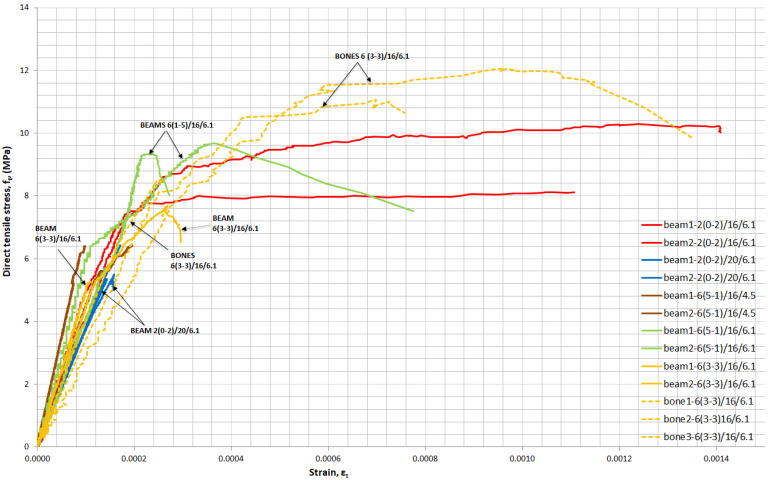
Stress–strain curves in direct tension—the influence of the fibre and shape of the specimens.

As mentioned before, beams from all mixtures and dogbones from mixtures with 6% fibres by volume, of which 3% were 13 mm long and 3% were 6 mm long, were tested in direct tension. In Figure 13, the influence of the specimen shape, quantity of superplasticizer, quantity, and length of fibres is illustrated. UHPC failed at first cracking, presenting only a linear elastic region. In contrast, most of the UHPFRC curves had postcracking regions, except the mixtures with a reduced amount of superplasticizer 6(5-1)/16/4.5 and increased water 2(2-0)/20/6.1. These two mixtures had brittle failure and maximum direct tensile stress of 6.1 and 6.3 MPa on average, respectively. 

**Figure 14:**
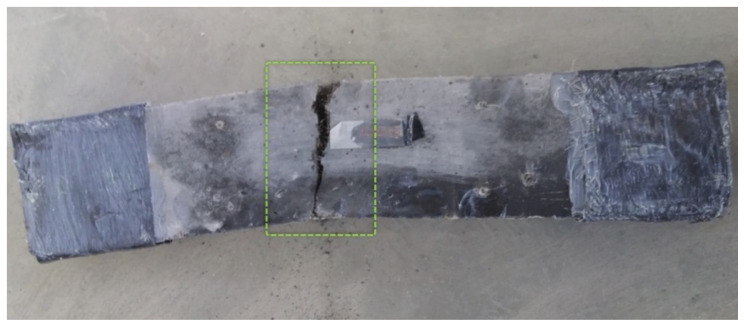
Failure of beam (direct tension test) from mixture 2(0–2)/16/6.1.

Both short and long fibres helped with direct tension by forming and bridging multi microcracks. Fibres hold both sides of the cracks and long fibres allow the crack width to extend and macrocracks to develop. The stress was maintained until fibres overcame their strength or pulled out from the matrix. Wille and Naaman [40] studied the pull-out behaviour of steel fibres and found out that straight fibres did not exhibit the same tensile strength and pulled out from the matrix with compressive strength up to 240 MPa. Increasing the strength of the matrix improved the bond strength and fibre effectiveness. The matrix in the mixtures of this research, 0/16/6.1, had a compressive strength of 124 MPa. Fibres pulled out and cracks propagated at the weakest point on the specimen. Finally, a large crack was formed, as illustrated in Figure 14, and the stress started to decrease. Long fibres contributed more than short fibres to carrying direct tension, although they caused a reduction in workability. Yu et al. [12] measured the slump flow of UHPFRC and noticed that, in mixtures with a combination of two types of steel fibres and when the percentage of short fibres was higher than that of long fibres, workability was higher. Workability is important for UHPFRC due to its influence on the fibre distribution in the specimens. The *w*/*b* reduction from 0.20 to 0.16 in the mixture with 2% by volume 13-mm-long fibres contributed to a significant increase in strain from 3.4 × 10^−3^ to 4.3 × 10^−3^ on average and an increase in ultimate tensile strength of 46.0%. The increased amount of superplasticizer led to an increase in strength and ductility. Concerning the beam specimens, mixtures 6(1-5)/16/6.1 and 6(3-3)/16/6.1 achieved, on average, an ultimate tensile strength of 9.5 and 6.5 MPa, respectively. However, the developed strain in the plastic region was low. When dogbone specimens were used for mixture 6(3-3)/16/6.1, the ultimate direct tensile strength reached 10.6 MPa on average and the strain hardening phase built up. In the literature, an equation is given to predict fibres per unit area *N_I_*, depending also on fibre orientation α, $N_{I}=\alpha \frac{V_{f}}{A_{f}}\;$(α = fibre orientation, *V_f_* = volume fraction of steel fibres in concrete, and *A_f_* = cross-sectional area of steel fibres). The specimen type has an influence on fibre orientation [36,41]. The orientation factor α is related to the boundaries (height and width of the specimen) and length of the fibres. The small dimensions of the middle cross section (b = 55 mm × h = 40 mm) of dogbone specimens are twice the fibre length but give an orientation factor of approximately 0.55, which is larger than the orientation factor of 0.41 for the beam specimen cross section (b = 50 mm × h = 100 mm) corresponding to 3D conditions. When the orientation factor increases, the number of fibres per unit area increases [36], as does, consequently, the direct tensile strength.

**Figure 15:**
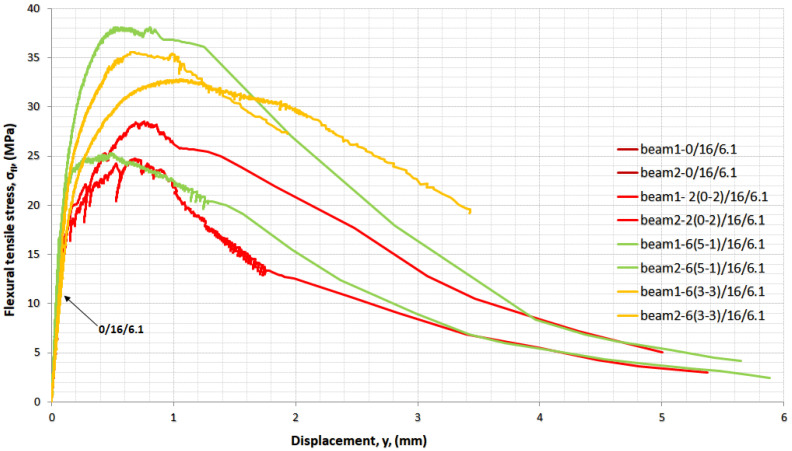
Stress–displacement curves in indirect tension—the influence of fibres.

In Figure 15, full stress–displacement curves from indirect tension (three-point bending) tests are shown. The modulus of elasticity from the flexural tensile test was, on average from two identical beams, 24, 30, 37, and 31 GPa for mixtures 0/16/6.1, 2(0-2)/16/6.1, 6(5-1)/16/6.1, and 6(3-3)/16/6.1, respectively. 

Curves for the mixtures without fibres 0/16/6.1 cannot be seen since they are behind the other graphs, failing in a brittle manner at 13.2 MPa and a corresponding displacement of 0.08 mm, on average. With the addition of 2% long fibres, the mixtures developed plastic deformations and exhibited high ductility. The ultimate tensile strength increased up to 26.6 MPa with a corresponding displacement of 0.72 mm, on average. The corresponding deflection at the ultimate flexure strength (on average) was 0.09 mm for mixture 0/16/6.1, 0.73 mm for mixture 2(0-2)/16/6.1, 0.53 mm for mixture 6(5-1)/16/6.1, and 0.86 mm for mixture 6(3-3)/16/6.1. Mixture 6(5-1)/16/6.1 with the use of a large amount of short fibres had the lower deflection at the ultimate flexural strength. Short fibres prevent the development of multi cracks and long length fibres do not allow crack width to extend and macro cracks to develop. A short fibre can bridge smaller cracks. When the long fibre increased from 1% to 3% per volume and the short fibre decreased from 5% to 3% per volume (mixture 6(3-3)/16/6.1) the deflection increased from 0.53 to 0.86 mm.

**Figure 16:**
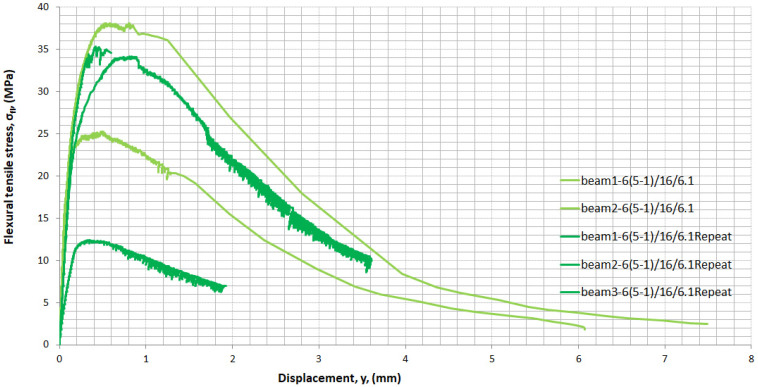
Stress–displacement curves in indirect tension from two mixtures of 6(5-1)/16/6.1.

The two beams from mixture 6(5-1)/16/6.1, with 6% by volume fibres, showed high scattering in the results. Beam specimens achieved an indirect tensile strength of 24.3 and 38.1 MPa with a difference of 13.80 MPa. Replacing 2% of short fibres with long fibres decreased the spread of results to 2 MPa and led mixture 6(3-3)/16/6.1 to achieve a flexural tensile strength of 34.2 MPa and associated displacement of 0.86 mm, on average. The spread of results from the mean is related to the size of the specimens. Large specimen cross sections, such as beams 100 mm × 100 mm, caused uneven fibre distribution and higher variability. Mixture 6(5-1)/16/6.1, a repeat mixture, was tested and the results are shown in Figure 16. It is important to note that high variability was once more observed in the results. The undesirable uneven distribution of fibres was caused not only due to the large section of the specimen but also due to the higher number of fibres. The number of fibres per 1 cm^3^ in mixture 2% (0-2) was 78 fibres of 13 mm long; in mixture 6% (5-1) it was 39 fibres of 13 mm long and 422 fibres of 6 mm long; and in mixture 6% (3-3) it was 117 fibres of 13 mm long and 253 fibres of 6 mm long. The mixture with 2% (0-2) had the lowest number of fibres and mixture 6(5-1) had the highest. A high dosage (high number) of steel fibres causes a decrease in workability [38]. In the mixture of 6% (5-1), 6-mm-long fibres were almost twice as numerous as in the mixture of 6% (3-3). The large section of the specimen allowed a 3D orientation of the fibres and that helped the fibres to clump together more easily. Short fibres clumped together more easily than long fibres and caused agglomerates of fibres and uneven distribution.

## 4. Finite Element Modelling of UHPFRC Mixture 6(3-3)/16/6.1

**Figure 17:**
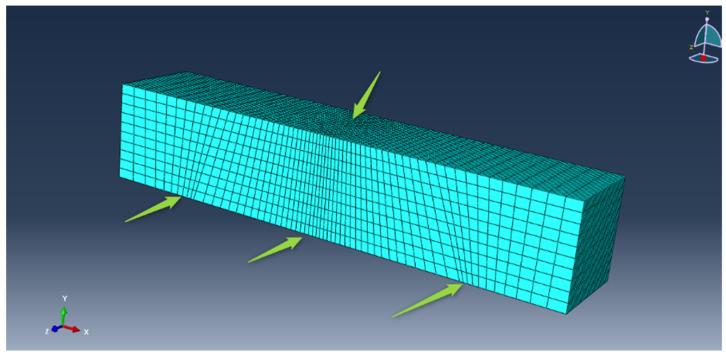
The 3D mesh of the specimen.

A finite element analysis (FEA), based on ABAQUS, is used for the simulation of the UHPFRC mixture 6(3-3)/16/6.1. A three-point bending test was executed through the displacement control on beams developed in ABAQUS. The geometry of the prismatic beam and the set-up of the model were the same as the indirect tension test of the experimental program. Reduced integration 20-noded brick elements with three degrees of freedom (C3D20R) were used to discretize UHPFRC beams. The 3D mesh was placed uniformly at the specimen. The density increased at the middle of the specimen and along the boundary areas to increase the accuracy of the results (Figure 17).

In this study, the concrete damage plasticity (CDP) model was applied, assuming that UHPFRC is a homogeneous material with a uniform distribution of the fibres in the matrix. The CDP model assumes that concrete responds to uniaxial loading by damage plasticity and the two fundamental failure mechanisms are tensile cracking and compressive crushing. The experimental data from cubes, cylinders, and dogbone specimens were used for the implementation of the CDP model. From the cylinder experimental test, the uniaxial stress–strain curve, modulus of Elasticity, and Poisson’s ratio were used. From the dogbone experimental test, the uniaxial stress displacement curve was used. Moreover, from the cube experimental data, density was used.

**Figure 18:**
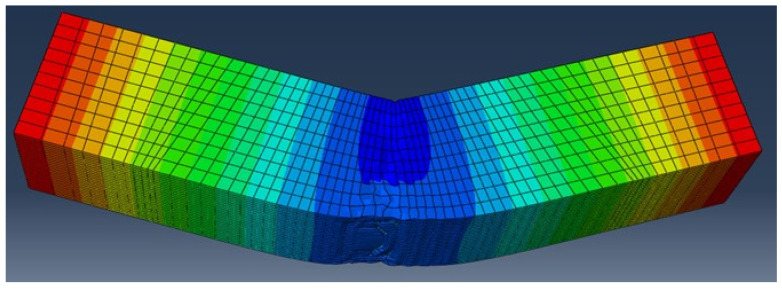
Contour diagrams of the displacement (along axis Y) of the specimen.

**Figure 19:**
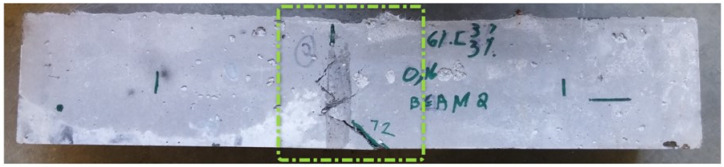
UHPFRC mixture 6(3-3)/16/6.1 specimen after failure in bending.

The distribution of the displacement (along axis Y) at the middle of the specimen coincides with the failure and deformed shape noted during the laboratory test (Figure 18 and Figure 19).

**Figure 20:**
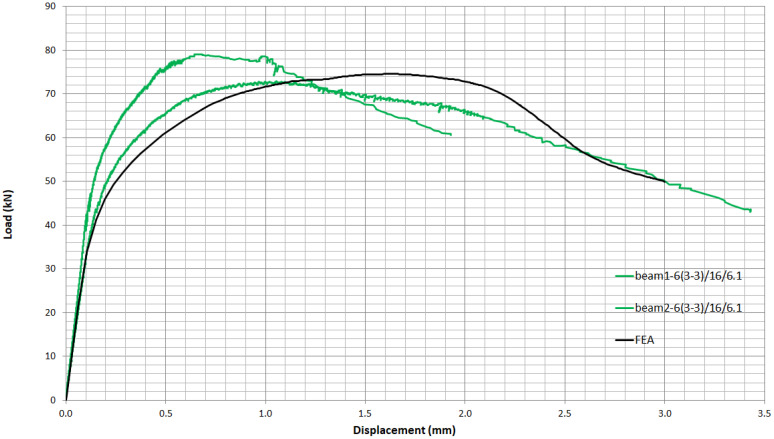
Comparison of load–displacement curves in indirect tension from experimental data and FEA.

To assess the validity of the finite element model, the results from the two curves of UHPFRC mixture 6(3-3)/16/6.1 beams, as shown in Figure 14, are compared with the results from FEA in ABAQUS. Figure 20 shows that the curves from experimental data and the finite element modelling are in good agreement. The slight differences may be attributed to the nonuniform fibre distribution in the tested specimens in contrast to the assumption of material homogeneity in the finite element analysis.

## 5. Conclusions

This study presents the findings of an extended experimental investigation that aimed to optimize the mechanical properties of UHPFRC in order to achieve higher resistance to projectile impact. From the findings of this research project, the following conclusions can be drawn:

- When the ratio of small to long steel fibres was altered to 3:3 rather than 5:1, the compressive strength of cubic specimens showed a slight improvement of 6.2% and the ductility increased with a large deformation developing in the plastic region.
- When the number of steel fibres increased from 2(0-2) to 6(5-1) by volume, the compression and direct and flexural tension increased by 13.4%, 3.6%, and 19.2%, respectively. Fibres’ increase contributed significantly to the increase in ultimate strain in compression from 4.3 × 10^−3^ to 5.6 × 10^−3^.
- The addition of 2% by volume steel fibres to the mixture increased by 25.6% and 23.3% the compressive strength for *w*/*b* 0.16 and 0.20, respectively, and the direct tensile strength by 70.0% for *w*/*b* 0.20% and 109.0% for *w*/*b* 0.16. The flexural strength exhibited the highest increase of 280.0% for *w*/*b* equal to 0.2% and 102.0% for *w*/*b* 0.16.
- The modulus of elasticity measured in compression testing was around 40 GPa. This is lower than expected for UHPFRC, which is attributed to the properties of the local materials.
- Poisson’s ratio, with an average of 0.25, was higher than the expected values for normal concrete, between 0.15 and 0.20.
- The cubic to cylindrical strength ratio was 0.88 for cubic specimens with a side length of 100 mm and cylindrical specimens with a diameter of 100 mm and height of 200 mm.
- The increase in the superplasticizer to binder ratio, from 4.5% to 6.1%, in mixture 6(5-1), helped in achieving a uniform distribution of fibres, due to the improved workability, and significantly improving the ductility in compression.
- Small specimens (dogbones) achieved a rather 2D orientation of the fibres, which contributed to the development of strain hardening behaviour during direct tension tests and increased the direct tensile strength from 6.5 to 10.6 MPa for mixture 6(3-3).
- A numerical simulation was developed using the CDP model in ABAQUS, which was cable of capturing the experimental three-point bending response of the UHPFRC 6(3-3) mixture.

Overall, mixture 6(3-3)/16/6.1 exhibited the best mechanical properties, achieved a compressive strength higher than 150 MPa, and presented great deformability in the plastic region, which are qualities needed for achieving the best response to an impact load. This mixture is expected to have the best performance under projectile impact loading. Indeed, in the extensive research, which examined the performance of the mixtures under projectile impact, the optimized mixture exhibited the best impact resistance [42].

## Figures and Tables

**Table 1:** Mixture designs for UHPC.

Materials (kg/m^3^)	0/16/4.5	0/16/6.1	0/20/6.1
Cement	880	880	880
Microsilica	220	220	220
Steel fibres 6 mm	0	0	0
Steel fibres 13 mm	0	0	0
Local sand 125–250 μm	475	475	475
Local sand 250–500 μm	358	358	358
Water	176	176	220
Superplasticizer	50	67	67

**Table 2:** Mixture designs for UHPFRC.

Materials (kg/m^3^)	2(0-2)/16/6.1	2(0-2)/20/6.1	6(5-1)/16/4.5	6(5-1)/16/6.1	6(3-3)/16/6.1
Cement	880	880	880	880	880
Microsilica	220	220	220	220	220
Steel fibres 6 mm	0	0	400	400	240
Steel fibres 13 mm	160	160	80	80	240
Local sand 125–250 μm	475	475	475	475	475
Local sand 250–500 μm	358	358	358	358	358
Water	176	220	176	176	176
Superplasticizer	67	67	50	67	67

**Table 3:** Number and dimensions of specimens.

Mix Design.	Number of Specimens				
	Cubes (100 mm × 100 mm × 100 mm)	Cylinders (d = 100 mm, h = 200 mm)	Prismatic Beams (100 mm × 50 mm × 500 mm)	Prismatic Beams (100 mm × 100 mm × 500 mm)	Dogbones
0/16/4.5	5	2	4	-	-
0/16/6.1	6	3	2	2	-
0/20/6.1	4	3	2	2	-
2(0-2)/16/6.1	6	3	2	2	-
2(0-2)/20/6.1	6	3	2	2	-
6(5-1)/16/4.5	5	2	4	-	-
6(5-1)/16/6.1	6	3	2	3	-
6(3-3)/16/6.1	6	3	2	3	3

**Table 4:** Test on each type of specimen.

Cubes (100 mm × 100 mm × 100 mm)	Cylinders (d = 100 mm, h = 200 mm)	Prismatic Beams (100 mm × 50 mm × 500 mm)	Prismatic Beams (100 mm × 100 mm × 500 mm)	Dogbones
Compression	Compression	Direct tension	Flexural tension	Direct tension

**Table 5:** Mechanical properties.

Mix Design	Compression Test				Direct Tension Test				Flexural Tension Test
	Compression Strength from Cubes (MPa)	Compression Strength from Cylinders (MPa)	E (GPa)	ν	Direct Tensile Strength from Beams (MPa)	Direct Tensile Strength from Bones (MPa)	Ε (GPa)	ν	Flexural Tensile Strength from Beams (MPa)
0/16/4.5	131.6	125.3	34.2	0.24	4.2	---	35.2	0.25	---
0/16/6.1	124.0	131.3	33.1	0.25	4.4	---	35.3	0.24	13.2
0/20/6.1	104.4	70.8	28.3	0.26	3.7	---	34.1	---	7.6
2(0-2)/16/6.1	155.8	135.3	35.2	0.22	9.2	---	35.4	0.25	26.6
2(0-2)/20/6.1	127.7	107.6	31.0	0.26	6.3	---	38.2	0.27	29.0
6(5-1)/16/4.5	163.8	147.7	41.4	0.22	6.1	---	47.8	0.25	---
6(5-1)/16/6.1	176.7	158.4	37.5	0.26	9.5	---	45.2	0.27	31.7
6(3-3)/16/6.1	187.6	167.1	38.6	0.25	6.5	10.6	43.5	0.24	33.3

## Data Availability

Not Applicable.
